# When Functional Assessment Meets Intravascular Imaging in Patients with Coronary Artery Disease †

**DOI:** 10.3390/jcdd12080319

**Published:** 2025-08-20

**Authors:** Grigorios Tsigkas, Kassiani-Maria Nastouli, Anastasios Apostolos, Panagiota Spyropoulou, Maria Bozika, Michail I. Papafaklis, Stella Rouzi, Effrosyni Tsimara, Antonios Karanasos, Virginia Mplani, Periklis Davlouros

**Affiliations:** 1 Department of Medicine, Division of Cardiology, University Hospital of Patras, 26504 Patras, Greece; gregtsig@upatras.gr (G.T.); kassienmarie@gmail.com (K.-M.N.); mariabozika29@gmail.com (M.B.); m.papafaklis@yahoo.com (M.I.P.); stella20022012@gmail.com (S.R.); eftsimara@gmail.com (E.T.); akaranasos@upatras.gr (A.K.); pdav@upatras.gr (P.D.); 2 Department of Cardiology, Harefield Hospital, Royal Brompton and Harefield Hospitals, Guy’s and St Thomas’ NHS Foundation Trust, London UB9 6JH, UK; 3 First Department of Cardiology, Medical School, National and Kapodistrian University, Hippocration Hospital of Athens, 11527 Athens, Greece; 4 Third Department of Internal Medicine, “Sotiria” General Hospital of Thoracic Diseases, 11527 Athens, Greece; pennyspyrop@gmail.com; 5 Intensive Care Unit, General University Hospital of Patras, 26504 Patras, Greece; virginiamplani@yahoo.gr

**Keywords:** Percutaneous Coronary Intervention, fractional flow reserve, intravascular imaging, coronary artery disease, optical coherence tomography, intravascular ultrasound

## Abstract

Percutaneous Coronary Intervention (PCI) has advanced significantly with the incorporation of imaging and physiology assessment techniques. Fractional Flow Reserve (FFR) and Non-Hyperemic Pressure indices (NHPIs) provide information regarding the functional significance of coronary lesions, while Intravascular Ultrasound (IVUS) and Optical Coherence Tomography (OCT) enhance anatomical characterization and guide stent implantation. This review explores the implementation of physiology- and imaging-guided strategies in clinical practice, comparing their efficacy and limitations. Novel technologies now allow for physiology estimation without hyperemic agents, and hybrid techniques, such as OCT-derived FFR, are increasingly integrated into clinical practice. These approaches offer the combined advantages of functional assessment and detailed anatomical imaging.

## 1. Introduction

An accurate assessment of coronary artery disease (CAD) is essential for optimal patient management in the catheterization laboratory. While coronary angiography remains the cornerstone for visualizing coronary anatomy, it has inherent limitations, particularly in evaluating intermediate lesions (40–70% stenosis). These lesions often pose a clinical dilemma, as their anatomical or functional significance cannot be reliably determined based on angiographic appearance alone. To overcome these limitations, wire-based physiological techniques and intravascular imaging modalities have emerged as essential tools in guiding clinical decision-making.

Fractional Flow Reserve (FFR) and Non-Hyperemic Pressure indices (NHPIs), like instantaneous wave free ratio (iFR) and resting full-cycle ratio (RFR) represent the leading functional methods to assess whether a lesion can cause ischemia [1]. Intravascular ultrasound (IVUS) and optical coherence tomography (OCT) represent the leading imaging tools used to clarify the significance of intermediate lesions [2,3]. In recent years, newer technologies have been developed. Quantitative flow ratio (QFR) or coronary angiography-guided FFR (FFRangio) are wire-free techniques which estimate physiological significance without the need for a hyperemic agent, while newest hybrid technologies are being developed, which combine imaging and physiological assessment.

The purpose of this paper is to provide an overview of currently available imaging and functional technologies used in Percutaneous Coronary Intervention (PCI), examines data concerning the indication for their use in clinical practice and a comparison between them in clinical practice, as well as their limitations, while discusses future integration of a hybrid approach, with technologies combining both imaging as well as physiological technologies.

## 2. Modalities for Functional Assessment

### 2.1. Fractional Flow Reserve (FFR)

The most frequently utilized functional modalities in PCI guidance are FFR and NHPIs [1]. While FFR remains a gold standard for assessing intermediate stenoses, its use should be tailored to individualized factors, lesion characteristics, and clinical presentation. FFR is defined as the ratio of the mean distal coronary pressure (Pd) to the mean aortic pressure proximal to a coronary stenosis (Pa) during conditions of maximal hyperemia, typically induced with pharmacological agents (e.g., with adenosine). FFR in a normal coronary artery equals 1. An FFR value of less than 0.75 is widely regarded as indicative of functionally significant coronary artery disease, warranting consideration for revascularization, whereas values above 0.80 are generally associated with the absence of myocardial ischemia and can be managed conservatively. The interval between 0.75 and 0.80 constitutes an indeterminate, or “gray,” zone in which clinical judgment is required to determine the most appropriate management strategy, integrating both anatomic and physiological data as well as patient-specific factors. Considerations regarding the use of FFR include the need for adenosine or other vasodilators to induce maximal hyperemia, which may lead to patient discomfort (such as chest pain, dyspnea, or flushing). While FFR is a useful tool for the estimation of ischemia, its use is limited in healthcare systems. Some of the reasons for its restricted use involve the further cost and time of the procedure, along with the aforementioned considerations [4].

### 2.2. Non-Hyperemic Pressure Indices (NHPIs)

NHPIs have been developed as alternatives and include iFR, diastolic hyperemia-free-ratio (DFR), RFR and resting whole-cycle Pd/Pa [5]. iFR is the only index of functional assessment alternative to FFR that has been tested in randomized clinical trials, and it demonstrates good correlation with FFR. The technique quantifies the ratio of distal coronary pressure (Pd) to aortic pressure (Pa) under resting conditions, without necessitating the administration of hyperemic agents. According to the Functional Lesion Assessment of Intermediate Stenosis to Guide Revascularization (DEFINE FLAIR) trial and the iFR Swedish Web—System for Enhancement and Development of Evidence-Based Care in Heart Disease Evaluated According to Recommended Therapies (SWEDHERT) trial, revascularization was indicated using cut-off values of 0.80 for FFR and <0.89 for iFR, while both methods demonstrated similar incidence of major adverse cardiac events (MACE) at 1-year follow up [6,7,8]. iFR was associated with lower rates of periprocedural chest pain and shorter procedural time, although both trials were confined by the relatively short follow-up period. RFR is an adenosine-free NHPI that calculates the lowest average value of Pd/Pa during an entire cardiac cycle and uses a cut-off of 0.89 with high diagnostic accuracy for severe lesions, a value that correlates highly with the 0.80 cut-off of FFR [9,10]. Resting whole cycle Pd/Pa is the ratio of the distal coronary blood pressure to the aortic pressure over the entire cardiac cycle and according to RESOLVE study, it utilizes a cut-off of 0.92 which is in agreement with FFR’s 0.80 for the estimation of lesion severity [11]. According to Shiode et al. iFR and resting cycle Pd/Pa can be combined for the optimization of the diagnostic accuracy in specific lesions [12]. Novel NHPIs demonstrate excellent performance compared with iFR. Despite the technical differences, dFR and iFR perform equivalently [13]. According to the latest European Society of Cardiology/European Association of Cardiothoracic Surgery (ESC/EACTS) guidelines, FFR and iFR have a class IA for the assessment of hemodynamic relevance of intermediate-grade lesions, when evidence of ischemia is not available [14]. FFR-guided PCI should be considered in patients with multivessel disease undergoing PCI. Similarly, in the latest American College of Cardiology/American Heart Association (ACC/AHA) guidelines, both modalities are recommended with a class IA for decision guidance in cases of angiographically intermediate stenoses, angina or an angina equivalent or undocumented ischemia [15].

## 3. Non-Invasive Physiology Assessment

Newer non-invasive techniques for functional assessment, such as QFR, FFRangio, FFR2D and FFR_CT_ derived from Computed Tomography Angiography (CTA) emerging as promising alternatives to traditional wire-based FFR [16,17,18,19,20]. These imaging-based methods use angiographic data and computational models to estimate coronary physiology without the need for pressure wire or hyperemic agents. These techniques offer a faster, less invasive, and more patient-friendly approach, potentially improving workflow in the catheterization lab and expanding access to physiological assessment in clinical practice.

## 4. Intravascular Modalities

### 4.1. Intravascular Ultrasound (IVUS)

IVUS is a contrast-free intravascular imaging technique, which employs sound technology in order to provide a 3D depiction of the arterial, perivascular structures, plaque extension and morphology. It aids in selecting appropriate stent size, avoiding stent malapposition or underexpansion, reaching an increased minimal stent area, providing better outcomes and detecting in-stent restenosis, dissections and hematomas [2,21,22]. IVUS has provided new insight into PCI guidance, influencing peri-procedural decisions and outcomes. Current European and American guidelines, recommend IVUS with class IIA for the assessment of intermediate unprotected left main stem lesions, complex coronary lesions and determination of stent restenosis mechanisms [14,15]. Moreover, IVUS may be considered as a reasonable option in non-left main (LM) stenting of intermediate stenoses with a class IIB recommendation.

### 4.2. Optical Coherence Tomography (OCT)

OCT utilizes low coherence (1300 μm) to produce intravascular images [3]. Two different techniques have been developed. The Time Domain OCT technique utilizes an imaging guide wire and an occlusion balloon to block blood flow. Frequency Domain OCT (FD-OCT) systems that include Fourier Domain OCT (FD-OCT), swept-source OCT (SS-OCT), or optical frequency-domain imaging (OFDI) do not require total blood clearance and the use of a balloon. Instead, a contrast agent flushes through the guiding catheter and removes blood flow, thus facilitating faster pullback speeds and image acquisition rates [23]. According to the ESC guidelines, OCT should be considered in patients to understand the mechanism behind stent failure (Class IIA) and for stent implantation optimization (Class IIB), as an alternate of IVUS [24].

## 5. Functional Assessment Versus Angiography

In the latest years several studies have been conducted comparing FFR with angiography. The Fractional Flow Reserve versus Angiography for Multivessel Evaluation (FAME) study was the cornerstone study that established the role of FFR in PCI guidance and compared clinical outcomes of FFR-guided versus angiography-guided PCI [25]. Patients were enrolled if they had multivessel disease meaning at least 50% stenosis in at least two or three major epicardial vessels. Clinical outcomes were significantly improved in the FFR-guided PCI group in comparison with the angiography-guided group [26]. FFR guidance has shown favorable long-term outcomes compared to medical therapy alone. In the FAME II study, FFR guidance in PCI plus optical medical therapy (OMT) was compared to OMT alone and proved to be superior in patients with stable coronary disease with at least one stenosis of at least 50% diameter reduction [27]. The main cause was the reduction in the need for urgent revascularization in the context of acute coronary syndrome. The FFR plus OMT group demonstrated more favorable clinical endpoints and angina symptoms improvement [28]. A comprehensive presentation of all the studies, comparing the aforementioned methods, is included in Table 1.

Flow Evaluation to Guide Revascularization in Multivessel ST-Elevation Myocardial Infarction (FLOWER-MI) trial randomly assigned, STEMI patients and multivessel disease who had already undergone revascularization of the culprit artery, to complete revascularization either by the guidance of FFR or by the guidance of angiography [29]. The outcomes showed that the FFR-guided group had a higher rate than the angiography-guided group, for the primary outcome, which was nonfatal myocardial infarction (MI), death by any cause or unplanned rehospitalization that led to revascularization at 1 year, a difference which was not statistically significant so that we can draw reliable conclusion. (HR: 1.32, 95% CI: 0.78–2.23, *p* = 0.31).

Similarly, Functional Testing Underlying coronary Revascularization (FUTURE) trial evaluated the role of FFR-guided treatment decision-making versus angiography-guided, in patients with multivessel disease [30]. Although the study was finished earlier, the 1 year follow up showed no significant difference between the two groups, regarding the primary endpoint of death, myocardial infarction, stroke or unplanned revascularization (4.6% in the FFR vs. 14.4% in angiography group, HR: 0.97; 95% CI: 0.69–1.36, *p* = 0.85). All-cause mortality was higher in the FFR group (3.7% vs. 1.5%, HR: 2.34, 95% CI: 0.97–5.68, *p* = 0.85), though again this outcome that was not statistically significant. Additionally, the premature cessation of the trial did not allow the detection of definitive mortality harm or benefit, though the intention-to-treat analysis at 1 year did not confirm this outcome.

In the RIPCORD II (Does Routine Pressure Wire Assessment Influence Management Strategy at Coronary Angiography for Diagnosis of Chest Pain?) study 1100 patients which underwent invasive coronary angiography were randomized into two groups, those undergoing angiography alone and those that angiography plus FFR assessment [31]. The outcomes analyzed were quality of life and hospital costs. According to their outcomes no significant difference was observed between the two arms (*p* = 0.88 for quality of life and *p* = 0.137 for the median hospital costs). The study did not demonstrate a significant improvement in either healthcare costs or quality of life outcomes for the FFR-guided group compared with angiography alone. It is important to note, however, that the trial was terminated prematurely (earlier than 1 year) following concerns regarding a potential increase in mortality observed in one study arm, which may have influenced the ability to fully assess the long-term clinical and economic impact of the intervention.

AQVA II (Physiology Optimized versus Angiography-Guided PCI) trial which compared the two modalities in complex high risk indicated procedures, showed that the FFR guided group presented a more optimal outcome in the post FFR values in comparison to the angiography only group. (77% in the physiology group and 84% in the angiography group, *p* < 0.0001) [32]. Additionally, in their subgroup analysis angiography derived- FFR was noninferior to microcatheter derived FFR (79% vs. 75%, respectively, *p* < 0.01 for noninferiority).

Recently, the data of the 5-year follow-up of FAME-3 study were published [33]. The 1-year follow-up of the trial showed that FFR was unable to reach non inferiority in comparison with Coronary Artery Bypass Grafting (CABG), suggesting that CABG remains a superior approach to three vessel disease (10.6% PCI vs. 6.9% CABG; HR: 1.5; 95% CI: 1.1–2.2, *p* = 0.35 for noninferiority). The 5-year follow-up showed no statistical significance in death or stroke, but a higher incidence of myocardial infarction (MI) with PCI (8% vs. 5% in CABG group, HR: 1.57, 95% CI: 1.04–2.36) and higher repeat revascularization rate (16% vs. 8%; HR: 2.02, 95% CI: 1.46–2.79) [34]. Although the differences between PCI and CABG have narrowed, CABG still demonstrates advantages, specifically in long term stability and reduced revascularization needs.

**Figure 1:**
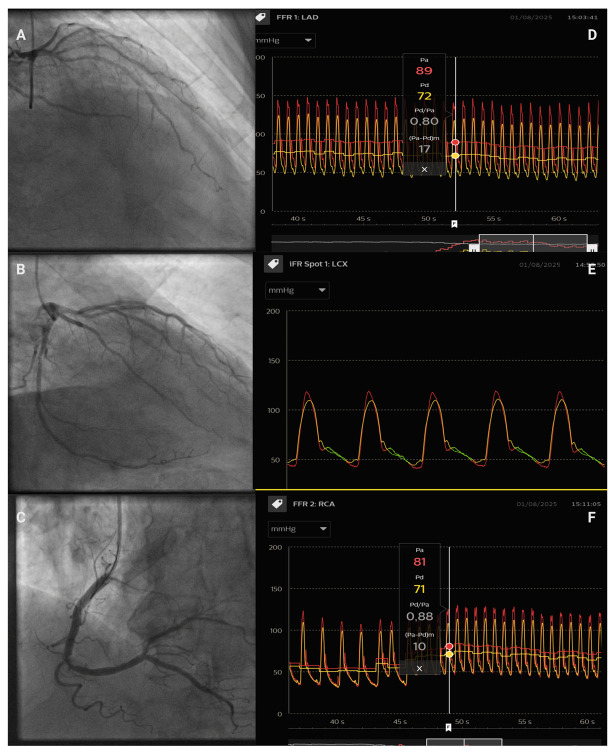
A case of multivessel disease guided by FFR/iFR assessment. (**A**) Angiographic image of left anterior descending artery (LAD). Pressure-wire pullback in the LAD revealed diffuse disease without a discrete, hemodynamically significant focal stenosis. (**B**) Angiographic image of left circumflex (LCX) artery. (**C**) Angiographic image of right coronary artery (RCA). (**D**) FFR in LAD with a borderline value 0.80. (**E**) iFR in LCX with a value of 0.98. (**F**) FFR in RCA with a normal value of 0.88.

In a recent case from our team (Figure 1), the patient had multivessel coronary disease. Angiography demonstrated diffuse atherosclerotic involvement of the left anterior descending (LAD), left circumflex (LCX), and right coronary (RCA) arteries. Physiologic assessment with FFR and iFR was performed in all three vessels: values were normal in the RCA and LCX, whereas both indices were borderline in the LAD (FFR 0.80 and iFR 0.87). Pressure-wire pullback in the LAD revealed diffuse disease without a discrete, hemodynamically significant focal stenosis; therefore, no percutaneous intervention was undertaken.

**Table 1:** Studies comparing FFR with angiography.

Trial Name of First Author Name (Date)/N	Study Design	Primary Endpoints	Principial Findings
FAMOUS NSTEMI (2015)/350 [35]	Randomized, FFR vs. angiography-guided management in NSTEMI patients	- Difference in the percentage of patients allocated to medical management. - MACE at 1-year follow-up.	- More patients initially received medical therapy with FFR guidance (22.7% vs. 13.2%, 95% CI: 1.4–17.7%, *p* = 0.022). - Revascularization was lower in the FFR group at 12 months (79.0% vs. 86.8%, absolute difference: 7.8%, 95% CI: −0.2% to 15.8%, *p* = 0.054).
FAME 5-YEAR FOLLOW-UP (2015)/1220 [26]	Randomized, FFR- vs. angiography-guided PCI in patients with multivessel coronary artery disease.	- MACE at 1-year follow-up and extended to 5-year follow-up.	- Fewer stents implanted per patient in the FFR-guided group (1.9 vs. 2.7, *p* < 0.0001).
FARGO (2018)/100 [36]	Randomized, FFR- vs. angiography-guided CABG in patients with coronary artery disease	Graft patency and clinical outcomes at 6-month follow-up.	- Similar rates of death, myocardial infarction, and stroke between groups (*p* = 0.64). - Mean FFR significantly decreased in deferred lesions from index to 6 months (0.89 ± 0.05 to 0.81 ± 0.11, *p* = 0.002). - The number of anastomoses per patient was lower in the FFR-guided group (2.6 ± 0.9 vs. 3.0 ± 0.9, *p* = 0.005). - Graft failure rates were similar for arterial vs. venous grafts (14.5% vs. 14.3%, *p* = 0.97).
GRAFFITI/172(2019) [37]	Randomized, FFR- vs. angiography-guided CABG in patients with coronary artery disease.	Graft Patency at 1 year follow-up.	- The FFR-guided group had fewer anastomoses compared to the angiography-guided group (2 [2; 3] vs. 3 [3; 3], *p* = 0.004). - The FFR-guided group had a significantly higher rate of functionally appropriate targeted grafting (79% vs. 68%, *p* = 0.008). - The FFR-guided group had a higher proportion of off-pump surgery (31% vs. 14%, *p* = 0.010) and minimally invasive surgery (10% vs. 2%, *p* = 0.036).
FLOWER-MI (2021)/1171 [29]	Randomized, FFR- vs. angiography-guided PCI in patients with STEMI and multivessel disease.	Composite of death from any cause, nonfatal myocardial infarction, or unplanned hospitalization leading to urgent revascularization at 1 year.	- Death occurred in 1.5% of the FFR group vs. 1.7% of the angiography group (HR: 0.89, 95% CI: 0.36–2.20). - Nonfatal myocardial infarction occurred in 3.1% vs. 1.7% (HR: 1.77, 95% CI: 0.82–3.84). - Unplanned hospitalization leading to urgent revascularization was similar (2.6% vs. 1.9%, HR: 1.34, 95% CI: 0.62–2.92). - Mean number of stents placed was lower in the FFR-guided group (1.01 ± 0.99 vs. 1.50 ± 0.86, *p* < 0.001).
FAME 3 (2021)/1500 [33]	Multicenter, randomized noninferiority trial comparing FFR-guided PCI using current-generation zotarolimus-eluting stents vs. CABG in patients with three-vessel coronary artery disease.	Composite of death from any cause, MI, stroke, or repeat revascularization at 1 year (noninferiority endpoint)	- FFR-guided PCI did not meet criteria for noninferiority compared to CABG (10.6% vs. 6.9%; HR: 1.5, 95% CI: 1.1–2.2, *p* = 0.35 for noninferiority). - Rates of death, MI, or stroke were not significantly different (7.3% vs. 5.2%; HR: 1.4. 95% CI: 0.9–2.1).
FUTURE (2021)/927 [30]	Randomized, FFR vs. angiography-guided management in patients with multivessel disease.	Composite of death, nonfatal MI, stroke, or unplanned revascularization at 1 year follow-up.	- All-cause mortality was higher in the FFR group (3.7% vs. 1.5%; HR: 2.34, 95% CI: 0.97–5.18, *p* = 0.06), leading to early termination of the study. - Stroke was less frequent in the FFR group (0.2% vs. 1.5%; HR: 0.13, 95% CI: 0.02–1.07, *p* = 0.06). - More patients were treated with medical therapy alone in the FFR group (17% vs. 9%, *p* = 0.002), reducing revascularization rates. - Study was prematurely stopped due to safety concerns (higher mortality in FFR-guided group).
RIPCORD-II (2022)/1100 [31]	Randomized FFR vs. angiography in patient with NSTEMI or stable angina, undergoing angiography.	National Health Service (NHS) hospital costs and quality of life at 1 year follow-up.	-No significant difference in median hospital costs between groups (£4510 vs. £4136, *p* = 0.137). - No difference in median quality of life scores (EuroQol EQ-5D-5L) (75 vs. 75, *p* = 0.88). - No difference in MACCE at 1 year (8.7% vs. 9.5%, *p* = 0.64). - No difference in individual clinical events: death (0.9% vs. 1.5%), stroke (1.5% vs. 2.2%), myocardial infarction (5.4% vs. 5.7%), and unplanned revascularization (8.7% vs. 9.5%). - FFR guidance did not result in cost savings or clinical benefit compared to angiography alone - Early termination of the study due to increased mortality rates in 1 arm.
AQVA-II (2024)/305 [32]	Randomized FFR vs. angiography-guided PCI in complex high-risk indicated procedures.	Post-PCI FFR value > 0.86.	- Higher rate of optimal post-PCI FFR in physiology-guided PCI (77% vs. 54%, absolute difference: 23%, *p* < 0.0001). - No difference between angiography-derived vs. microcatheter-derived FFR for PCI guidance (*p* < 0.01, confirming noninferiority). - Contrast dye volume was significantly higher in the angiography-guided group (170 mL vs. 140 mL, *p* < 0.0001).

## 6. Intravascular Imaging Versus Angiography

Intravascular imaging modalities have been useful tools to surpass limitations of Quantitive Coronary Angiography (QCA) and guide PCI decision-making [38]. Okamura et al. found that OCT produces smaller measurements of minimum lumen area than IVUS and offers clearer visualization of some intra-stent features, while Habara et al. showed that IVUS guidance is superior to OCT guidance for achieving optimal stent expansion, minimizing residual plaque at stent edges, and visualizing vessel borders. [39,40]. According to the Optical Coherence Tomography—Guided versus Angiography Guided PCI (ILUMIEN) trials, OCT was found to be non-inferior to IVUS for assessing stent expansion but superior in detecting malapposition and dissections [41,42]. Ramasamy et al. meta-analysis showed that for the detection of hemodynamically crucial lesions, OCT had higher specificity and accuracy than IVUS but similar sensitivity [43]. Recently iSIGHT (Optical Coherence Tomography versus Intravascular Ultrasound and Angiography to Guide Percutaneous Coronary Interventions) and ROCK II (a multidisciplinary Rehabilitation intervention for sudden Out-of-hospital Cardiac arrest survivors focusing on return to worK) trials validated that as for stent expansion guidance, OCT was non-inferior to IVUS and superior compared to angiography [44,45]. OCT/IVUS guidance showed that it reduced the adverse events in comparison to angiography guided PCI, especially in LM Disease. Both imaging modalities provide valuable assistance for intravascular imaging and OCT has proved to be a fundamental tool in lesion characterization, as well as in stent placement in bifurcation lesions and has an ever-increasingly important role in defining short- and long-term outcomes in PCI [46]. Further data are needed to demonstrate whether there is superiority of one method in different clinical settings. Completed and ongoing studies concerning OCT-, IVUS- and angiography-guided PCI are summarized in Table 2.

Recently, the RENOVATE-COMPLEX (Intravascular Imaging—Guided Complex PCI) study, a randomized controlled study that compared the efficacy of either IVUS or OCT in PCI in comparison to angiography found that IVUS-guided-PCI improved clinical outcomes compared to angiography-guided-PCI for complex lesions [47]. In the three-years-follow up, among 1639 patients, the primary endpoint of death from cardiac causes, clinically driven revascularization, target-vessel MI, occurred in 7.7% in the imaging group and 12.3% in the angiography group. (HR: 0.64, 95%, CI: 0.45–0.89, *p* = 0.008). Secondary outcomes were also improved. Target-vessel failure occurred in 5.1% of the IVUS-guided group and 8.7% in the angiography-guided-group (HR: 0.59, 95% CI = 0.39–0.90) and the composite of target-vessel-related-MI or cardiac death was 5.3% vs. 8.5%, respectively (HR: 0.63, 95%, CI: 0.42–0.93).

In ULTIMATE III (Intravascular Ultrasound vs. Angiography—Guided Drug—Coated Balloon Angioplasty) 260 high-bleeding risk patients with de novo coronary lesions were randomized to IVUS-guides drug coated balloon (DCB) angioplasty versus angiography guided [48]. According to their outcomes the IVUS-guided group showed a lower 7-month-in-segment-stent late lumen loss compared with angiography group (-0.10 mm ± 0.34 mm vs. 0.03 ± 0.52 mm, 95% CI: 0.02–0.26, *p* = 0.025), highlighting the importance of imaging in this sensitive patient population [49].

In a subgroup analysis of IVUS-ACS the investigators compared the efficacy of IVUS-guided PCI vs. angiography-guided PCI in Acute Coronary Syndrome (ACS) patients with diabetes [50]. At the follow up time, the primary end point, target-vessel-failure, was more frequently observed in the angiography-guided group, in comparison to the IVUS group (8.3% vs. 3.6%, respectively, HR: 0.46, 95%, CI: 0.27–0.81, *p* = 0.007). There was lower rate of target vessel revascularization in the IVUS group in comparison to the angiography group (0.9% vs. 3.8%, *p* = 0.003), IVUS reduced all-cause mortality (HR: 0.30, 95% CI: 0.10–0.93, *p* = 0.037) and reduced target vessel failure risk without MI during the procedure (2% vs. 6.7%, HR: 0.29, 95%, CI: 0.15–0.57, *p* < 0.001) The study showcased that a more detailed, imaging approach in the stent implantation procedure can improve revascularization outcomes and mortality simultaneously in this high-risk population group [51].

The European Trial on Optical Coherence Tomography Optimized Bifurcation Event Reduction (OCTOBER) trial investigated the application of OCT-guided versus angiography-guided revascularization in patients with complex coronary artery bifurcation lesions [52]. Among the patients in the OCT-guided group, the primary endpoint was significantly lower than those in the angiography-guided group (10.1% vs. 14.1%, respectively, HR: 0.70, 95% CI: 0.50–0.98, *p* = 0.035). Complications related to the procedure were similar between the groups, a fact suggesting that OCT-guidance improved outcomes without increasing the risk of complications.

OCT Guided Coronary Stent Implantation Compared with Angiography: A Multicenter Randomized Trial in PCI (ILUMIEN IV: OPTIMAL PCI) investigated the minimum stent area after PCI and target vessel failure at 2 years [53]. OCT-group showed favorable results regarding minimum stent area (5.72 ± 2.04 mm^2^ vs. 5.36 ± 1.87 mm^2^, *p* < 0.001) but did not differ in the target vessel failure at 2 years (*p* = 0.45).

Recently, the outcomes of a secondary analysis of the Optical Coherence Tomography-Guided or Intravascular Ultrasound-Guided Percutaneous Coronary Intervention (OCTIVUS) trial were presented [54]. OCTIVUS was a multicenter randomized controlled trial that led to the non-inferiority of OCT-guided PCI in comparison to IVUS-guided PCI in complex coronary lesions, regarding the composite of death from cardiac causes, target-vessel myocardial infarction, or ischemia-driven target-vessel revascularization at 12 months after the index procedure [55]. In the secondary analysis, the investigators aimed to further examine cardiovascular events with or without stent optimization in an IVUS-guided group versus an OCT-guided group. Optimization was successful in 51.6% of the population and the primary endpoint, TVF was observed more on the non-optimized group (HR: 0.52, 95% CI: 0.35–0.77, *p* < 0.001), with the benefit most pronounced in the OCT-Guided group, indicating that the use of optimization may offer better results for the patients.

**Figure 2:**
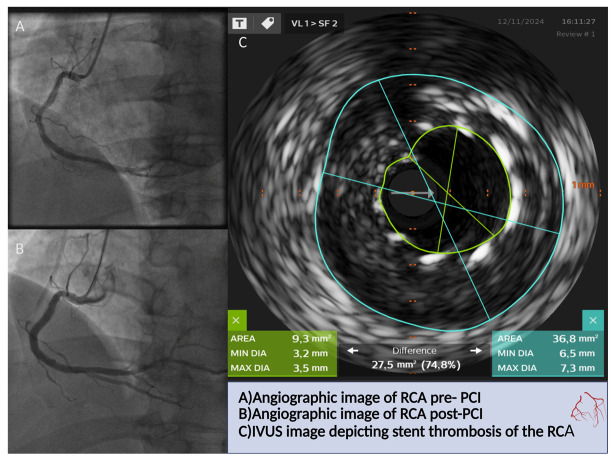
A case of stent thrombosis due to undersizing. (**A**) Initial angiographic image of Right coronary artery (RCA) pre- primary coronary intervention (PCI). (**B**) Final image of RCA post- PCI. (**C**) IVUS image depicting the stent thrombosis of RCA. Commas in the figure labels indicate the decimal point (e.g., 9,3 mm^2^ = 9.3 mm^2^).

**Figure 3:**
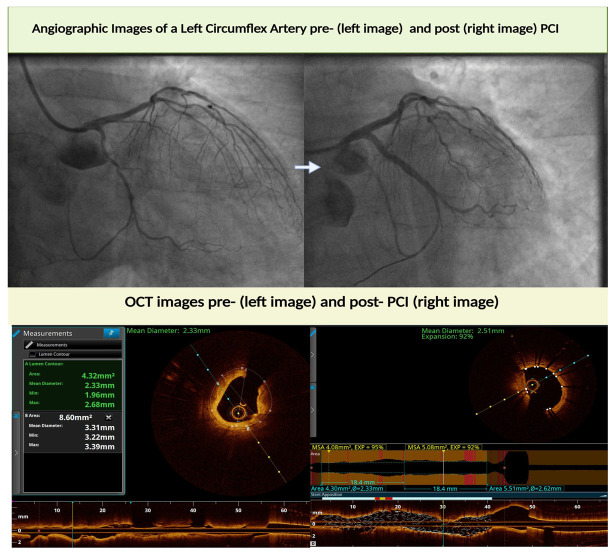
OCT-Guided-PCI for. (**A**) Upper left image: Initial angiographic imaging of left circumflex (LCX) artery; (**B**) Bottom left image: Initial OCT—image of the true dimensions of LCX; (**C**) Upper right: final angiographic image of LCX post PCI; (**D**) Bottom right: Final OCT-image-post PCI.

In Figure 2 and Figure 3, practical applications of intravascular imaging for PCI optimization in daily practice are presented. Figure 2 shows IVUS-guided management of stent thrombosis. IVUS identified stent undersizing/under-expansion, leading to selection and implantation of a correctly sized drug eluting stent. The initial and final angiographic outcomes are also presented. Figure 3 illustrates OCT-guided-PCI of the left circumflex artery. Angiography underestimated vessel caliber (approximately 1 mm) while OCT measured a diameter of 3.3 mm and defined plaque morphology. Two drug-eluting-stents were selected with optimal final expansion and apposition.

**Table 2:** Completed studies of OCT vs. IVUS vs. angiography guidance in PCI.

Trial Name or First Author Name (Date)/N	Study Design	Primary Endpoints	Principal Findings
HOME-DES IVUS (2010)/210 [56]	Randomized, IVUS- vs. angiography-guided DES implantation in patients with complex coronary artery disease.	- MACE at 18-month follow-up (composite of death, myocardial infarction, and reintervention).	- Stent thrombosis occurred in 3.8% (IVUS) vs. 5.7% (Angiography). - Higher rate of post-dilatation in IVUS-guided group (33% vs. 0%, *p* < 0.001). - Higher balloon inflation pressure in IVUS group (16.4 ± 1.7 vs. 15.2 ± 1.5 atm, *p* < 0.001). - Longer fluoroscopy time in IVUS group (12.6 ± 5.9 vs. 7.8 ± 3.6 min, *p* = 0.02). - Higher contrast volume in IVUS group (132.5 ± 43.5 vs. 112.5 ± 30.6 mL, *p* = 0.03).
Habara et al. (2012)/70 [40]	Randomized, FD-OCT vs. IVUS-guided PCI in patients with de novo coronary lesions.	Post PCI stent expansion assessed by IVUS.	- Minimum stent area was significantly smaller in the FD-OCT group compared to IVUS (6.1 ± 2.2 mm^2^ vs. 7.1 ± 2.1 mm^2^, *p* = 0.04). - Mean stent area was lower in FD-OCT (7.5 ± 2.5 mm^2^ vs. 8.7 ± 2.4 mm^2^, *p* = 0.04). - Focal stent expansion was significantly lower in FD-OCT (64.7% ± 13.7% vs. 80.3% ± 13.4%, *p* = 0.002). - Diffuse stent expansion was lower in FD-OCT (84.2% ± 15.8% vs. 98.8% ± 16.5%, *p* = 0.003). - Significant residual reference segment stenosis was more frequent in FD-OCT group (22.9% vs. 2.9%, *p* = 0.03).
CLI-OPCI (2012)/670 [57]	Retrospective observational study, comparing OCT vs. angiography-guided PCI.	Composite of cardiac death or MI at 1 year.	- Lower risk of cardiac death (1.2% vs. 4.5%, *p* = 0.010) and cardiac death or MI (6.6% vs. 13.0%, *p* = 0.006) in the OCT group. - The composite endpoint was significantly lower in the OCT group (9.6% vs. 14.8%, *p* = 0.044). - After multivariable analysis, OCT guidance was associated with a lower risk of cardiac death or MI (OR 0.49, 95% CI: 0.25–0.96, *p* = 0.037). - Propensity-score adjusted analysis confirmed the benefit of OCT-guided PCI (OR 0.37, 95% CI: 0.10–0.90, *p* = 0.050).
AVIO (2013)/284 [58]	Randomized, IVUS- vs. angiography-guided DES implantation in complex coronary lesions.	Post-procedure in-lesion MLD.	- IVUS-guided PCI resulted in significantly larger post-procedure MLD (2.70 ± 0.46 mm vs. 2.51 ± 0.46 mm, *p* = 0.0002). - Higher post-dilatation rates in IVUS-guided PCI (88.3% vs. 68.4%, *p* < 0.0001).
Kim et al. (2013)/543 [59]	Randomized, IVUS- vs. angiography-guided DES implantation in patients with long coronary artery stenoses.	MACE at 1 year (composite of cardiovascular death, MI, stent thrombosis, and target vessel revascularization).	- No significant difference in MACE between IVUS- and angiography-guided groups in the intention-to-treat analysis (4.5% vs. 7.3%, RR 0.59, 95% CI: 0.28–1.24, *p* = 0.16). - In the per-protocol analysis (excluding crossover patients), IVUS guidance was associated with a significantly lower MACE rate (4.0% vs. 8.1%, RR 0.48, 95% CI: 0.23–0.99, *p* = 0.048). - Post-procedural minimal lumen diameter was significantly larger in the IVUS-guided group in the per-protocol analysis (2.58 mm vs. 2.51 mm, *p* = 0.04) but not in the intention-to-treat analysis. - Adjunct post-dilation was more frequent in the IVUS group (54.6% vs. 44.5%, *p* = 0.03).
RESET IVUS (2013)/1574 [60]	Retrospective, observational study comparing IVUS- vs. angiography-guided DES implantation in short-length lesions.	MACE at 1 year (composite of cardiovascular death, MI, and TVR).	- Stent thrombosis was similar in both groups (0.2% vs. 0.2%, HR: 0.68, 95% CI: 0.06–7.52, *p* = 0.754).
AIR-CTO (2015)/230 [42]	Randomized, IVUS- vs. angiography-guided PCI in patients with chronic total occlusion.	In-stent LLL at 1-year follow-up.	- Lower LLL in the IVUS-guided group (0.28 ± 0.48 mm vs. 0.46 ± 0.68 mm, *p* = 0.025). - Lower in-true-lumen restenosis rate in IVUS-guided PCI (3.9% vs. 13.7%, *p* = 0.021). - Lower stent thrombosis rate in IVUS-guided PCI at 2 years (0.9% vs. 6.1%, *p* = 0.043). - Higher procedural success based on IVUS criteria in IVUS-guided PCI (91% vs. 68%, *p* = 0.024).
Tan et al. (2015)/123 [61]	Randomized, IVUS- vs. angiography-guided PCI in elderly patients with unprotected left main coronary artery stenosis.	MACE at 2 years (composite of death, nonfatal myocardial infarction, and TLR).	- Lower MACE rate in IVUS-guided PCI (13.1% vs. 29.3%, *p* = 0.031). - Lower TLR rate in IVUS-guided PCI (9.1% vs. 24.0%, *p* = 0.045). - Multivariable Cox proportional hazard model identified distal lesion as an independent predictor of MACE (HR: 1.99, 95% CI: 1.129–2.367, *p* = 0.043). - IVUS guidance was an independent factor for survival free of MACE (HR: 0.414, 95% CI: 0.129–0.867, *p* = 0.033).
CTO-IVUS (2015)/402 [62]	Randomized, IVUS- vs. angiography-guided PCI in patients with chronic total occlusion.	MACE at 12 months (composite of cardiac death, MI, or TVR).	- Lower MACE rate in IVUS-guided PCI (2.6% vs. 7.1%, HR: 0.35, 95% CI: 0.13–0.97 p = 0.035). - Lower composite of cardiac death or MI in IVUS-guided PCI (0% vs. 2.0%, *p* = 0.045). - Higher postprocedural minimum lumen diameter in IVUS-guided PCI (2.64 ± 0.35 mm vs. 2.56 ± 0.41 mm, *p* = 0.025). - Greater use of high-pressure post-stent dilation in IVUS-guided PCI (51.2% vs. 41.3%, *p* = 0.045).
ILUMIEN II (2015)/940 [41]	Observational, propensity-matched study comparing OCT- vs. IVUS-guided PCI.	Stent expansion (MSA divided by the mean of the proximal and distal reference lumen areas).	- OCT detected more post-PCI stent malapposition (26.6% vs. 13.6%, *p* = 0.0002), tissue protrusion (63.6% vs. 27.3%, *p* < 0.0001), and edge dissections (23.1% vs. 5.2%, *p* < 0.0001). - In-segment diameter stenosis was slightly higher in the OCT group (13.3% vs. 11.2%, *p* = 0.009).
CREDO/KYOTO AMI (2015)/3028 [63]	Observational registry study comparing IVUS- vs. angiography-guided PCI in STEMI patients undergoing primary PCI.	TVR at 5 years.	- Lower unadjusted TVR rate in IVUS-guided PCI (22% vs. 27%, *p* < 0.001), but no significant difference after adjustment (HR: 1.14, 95% CI: 0.86–1.51, *p* = 0.38). - Lower unadjusted definite stent thrombosis (1.2% vs. 3.1%, *p* = 0.003), but no significant difference after adjustment (HR: 0.58, 95% CI: 0.19–1.72, *p* = 0.33). - Higher final balloon inflation pressure in IVUS-guided PCI (15.6 ± 4.0 atm vs. 14.5 ± 3.6 atm, *p* < 0.001). - Longer door-to-balloon time in IVUS-guided PCI (*p* < 0.001).
DOCTORS (2016)/240 [64]	Randomized, OCT- vs. angiography-guided PCI in patients with non-ST-segment elevation acute coronary syndromes.	Functional results of PCI assessed by post-PCI FFR.	- Higher post-PCI FFR in OCT-guided PCI (0.94 ± 0.04 vs. 0.92 ± 0.05, *p* = 0.005). - More frequent poststent overdilation in OCT-guided PCI (43% vs. 12.5%, *p* < 0.0001). - Lower residual stenosis in OCT-guided PCI (7.0 ± 4.3% vs. 8.7 ± 6.3%, *p* = 0.01).
Zhang et al. (2016)/84 [65]	Randomized, IVUS- vs. angiography-guided DES implantation in small coronary artery lesions.	Post-procedure in-lesion MLD.	- Larger post-procedure MLD in IVUS-guided PCI (2.77 ± 0.19 mm vs. 2.53 ± 0.21 mm, *p* = 0.000). - Greater acute gain in IVUS-guided PCI (1.87 ± 0.28 mm vs. 1.63 ± 0.27 mm, *p* = 0.000). - Smaller final stenosis in IVUS-guided PCI (6.72 ± 2.56% vs. 7.94 ± 2.47%, *p* = 0.029).
Sheth/Kajander et al. (2016)/642 [66]	Propensity-matched cohort study, OCT- vs. angiography-guided PCI in STEMI patients (substudy of the TOTAL trial).	Composite of cardiovascular death, MI, stent thrombosis, and TVR at 1 year.	- Larger final MLD in OCT-guided PCI (2.99 ± 0.48 mm vs. 2.79 ± 0.47 mm, *p* < 0.0001). - Longer procedure time in OCT-guided PCI (median 58 min vs. 38 min, *p* < 0.0001). - Higher total contrast dose in OCT-guided PCI (239.7 ± 81.1 mL vs. 193.3 ± 78.6 mL, *p* < 0.0001).
ILUMIEN III (2016)/450 [67]	Randomized, OCT- vs. IVUS- vs. angiography-guided PCI.	Post-PCI MSA measured by OCT.	- Final median MSA (primary endpoint): OCT = 5.79 mm^2^ (IQR: 4.54–7.34), IVUS = 5.89 mm^2^ (4.67–7.80), Angiography = 5.49 mm^2^ (4.39–6.59). - OCT guidance was non-inferior to IVUS (one-sided 97.5% lower CI −0.70 mm^2^, *p* = 0.001), but not superior (*p* = 0.42). - Procedural MACE: OCT = 3%, IVUS = 1%, Angiography = 1% (*p* = 0.37 for OCT vs. IVUS, *p* = 0.37 for OCT vs. Angiography). - Greater stent expansion in OCT vs. Angiography (87.6% ± 16.6 vs. 82.9% ± 12.9, *p* = 0.02). - Lower rate of major untreated dissections in OCT vs. IVUS (14% vs. 26%, *p* = 0.009). - Lower rate of major untreated stent malapposition in OCT vs. IVUS (11% vs. 21%, *p* = 0.02).
OPINION (2018)/829 [68]	Randomized, non-inferiority study comparing OFDI vs. IVUS-guided PCI.	TVF at 12 months (composite of cardiac death, target-vessel myocardial infarction, and ischemia-driven target vessel revascularization).	- TVF occurred in 5.2% of OFDI-guided PCI vs. 4.9% of IVUS-guided PCI (HR: 1.07, upper one-sided 95% CI 1.80, *p*_non-inferiority = 0.042). - Binary restenosis at 8 months was similar between groups (in-stent: 1.6% vs. 1.6%, *p* = 1.00; in-segment: 6.2% vs. 6.0%, *p* = 1.00). - Higher contrast use in OFDI-guided PCI (164 ± 66 mL vs. 138 ± 56 mL, *p* < 0.001).
Jones et al. (2018)/87,166 [69]	Observational cohort study from the Pan-London PCI registry, comparing OCT- vs. IVUS- vs. angiography-guided PCI.	All-cause mortality at a median follow-up of 4.8 years.	- Lower mortality in OCT-guided PCI (7.7%) vs. IVUS-guided PCI (12.2%) vs. angiography-guided PCI (15.7%), *p* < 0.0001. - Multivariate Cox analysis: OCT vs. angiography-alone (HR: 0.48, 95% CI: 0.26–0.81, *p* = 0.001); OCT vs. IVUS (HR: 0.88, 95% CI: 0.61–1.38, *p* = 0.43). - Lower in-hospital MACE in OCT-guided PCI (1.3%) vs. IVUS (1.4%) vs. angiography (1.8%), *p* = 0.016. - Lower in-hospital mortality in OCT-guided PCI (0.3%) vs. IVUS (0.4%) vs. angiography (0.7%), *p* = 0.010. - Lower Q-wave MI in OCT-guided PCI (0.2%) vs. IVUS (0.5%) vs. angiography (0.7%), *p* = 0.046.
ULTIMATE (2018)/1448 [70]	Randomized, IVUS- vs. angiography-guided DES implantation in all-comer patients.	TVF at 12 months (composite of cardiac death, target vessel MI, and clinically driven TVR).	- Lower TVF rate in IVUS-guided PCI (2.9%) vs. angiography-guided PCI (5.4%), HR: 0.530, 95% CI: 0.312–0.901, *p* = 0.019. - Lower clinically driven TVR in IVUS-guided PCI (1.5% vs. 2.9%, HR: 0.514, 95% CI: 0.248–1.066, *p* = 0.07). - Lower definite/probable stent thrombosis in IVUS-guided PCI (0.1% vs. 0.7%, HR: 0.199, 95% CI: 0.023–1.704, *p* = 0.10).
IVUS-XPL 5-year follow-up (2020)/1400 [71]	Randomized, IVUS- vs. angiography-guided DES implantation in long coronary lesions	MACE at 5 years (composite of cardiac death, target lesion-related MI, or ischemia-driven TLR).	- Lower MACE rate in IVUS-guided PCI (5.6% vs. 10.7%, HR: 0.50, 95% CI: 0.34–0.75, *p* = 0.001). - Lower ischemia-driven TLR in IVUS-guided PCI (4.8% vs. 8.4%, HR: 0.54, 95% CI: 0.33–0.89, *p* = 0.007). - Lower cardiac death rate in IVUS-guided PCI (0.9% vs. 2.2%, HR: 0.43, 95% CI: 0.17–1.12, *p* = 0.074). - Lower MACE rate between 1 and 5 years in IVUS-guided PCI (2.8% vs. 5.2%, HR: 0.53, 95% CI: 0.29–0.95, *p* = 0.031).
iSIGHT (2021)/151 [45]	Randomized, OCT- vs. IVUS- vs. angiography-guided PCI.	Post-procedure stent expansion (minimum stent area ≥90% of the average reference lumen area).	- OCT guidance achieved stent expansion of 98.01 ± 16.14%, which was noninferior to IVUS (91.69 ± 15.75%, *p*_non-inferiority < 0.001) and superior to angiography (90.53 ± 14.84%, *p* = 0.041). - Lower rate of incomplete stent apposition in OCT-guided PCI (70.6%) compared to IVUS (92.2%) and angiography (81.1%), *p* = 0.016. - Higher proportion of optimal stent expansion (≥90%) in OCT (74.5%) vs. IVUS (49.0%) vs. angiography (50.9%), *p* = 0.014.
THE ROCK II (2021)/730 [44]	Multicenter, retrospective European study comparing OCT- vs. IVUS- vs. angiography-guided PCI for distal LM stenting	TLF at 1 year (composite of cardiac death, target vessel MI, and TLR).	- Lower TLF in intravascular imaging-guided PCI vs. angiography (12.7% vs. 21.2%, *p* = 0.039). - Propensity-matched analysis: TLF at 1 year: 16% (angiography), 7% (OCT), 6% (IVUS), *p* = 0.03 for IVUS/OCT vs. angiography. - TLF IVUS vs. angiography (HR: 0.37, 95% CI: 0.15–0.91, *p* = 0.03), OCT vs. angiography (HR: 0.43, 95% CI: 0.18–1.01, *p* = 0.05). - Lower TLR in OCT-guided PCI (4.3%) vs. IVUS (10.2%) vs. angiography (11.0%), HR for OCT vs. angiography 0.39, 95% CI: 0.18–0.85.
EROSION III (2022)/246 [72]	Randomized, OCT-guided vs. angiography-guided reperfusion in STEMI with early infarct artery patency.	Rate of stent implantation at 1 year follow up.	- Lower rate of stent implantation in OCT-guided PCI (43.8%) vs. angiography-guided PCI (58.8%), *p* = 0.024. - Lower residual angiographic diameter stenosis in OCT-guided PCI (8.7% ± 3.7%) vs. angiography-guided PCI (11.8% ± 4.6%), *p* < 0.001. - Higher rate of nonstenting treatment in OCT-guided PCI (56.2%) compared to angiography (41.2%).
HONEST (2022)/75 [73]	Randomized, OCT- vs. angiography-guided magnesium bioresorbable scaffold (MBRS) implantation in NSTEMI patients.	6-month healing stage As a result, to OCT.	- MBRS resorption at 6 months: OCT-guided 77.0% [IQR: 68.5–85.5] vs. angiography-guided 76.5% [IQR: 67.9–85.5], *p* = 0.97. - Reduction in MLA was greater in OCT-guided PCI (-2.3 ± 1.6 mm^2^ vs. -1.4 ± 1.4 mm^2^, *p* = 0.02). - Greater reduction in total lumen volume in OCT-guided PCI (−27.1 ± 32.5 mm^3^ vs. −5.0 ± 32.9 mm^3^, *p* < 0.01).
RENOVATE-COMPLEX (2023)/1620 [47]	Randomized, IVUS- or OCT-guided vs. angiography-guided PCI in complex coronary artery disease.	Composite of cardiac death, target-vessel myocardial infarction, or clinically driven target-vessel revascularization at a median follow-up of 2.1 years.	- Lower primary endpoint event rate in IVUS/OCT-guided PCI (7.7%) vs. angiography-guided PCI (12.3%), HR: 0.64, 95% CI: 0.45–0.89, *p* = 0.008. - Lower risk of cardiac death in IVUS/OCT-guided PCI (1.7% vs. 3.8%, HR: 0.47, 95% CI: 0.24–0.93). - Lower target-vessel-related MI in IVUS/OCT-guided PCI (3.7%) vs. angiography-guided PCI (5.6%), HR: 0.74, 95% CI: 0.45–1.22. - Lower target-vessel revascularization in IVUS/OCT-guided PCI (3.4%) vs. angiography-guided PCI (5.5%), HR: 0.69, 95% CI: 0.40–1.18.
ILUMIEN IV (2023)/2487 [53]	Randomized, OCT- vs. angiography-guided PCI in patients with diabetes or complex coronary lesions.	Minimum stent area post-PCI (assessed by OCT) - TVF at 2 years (composite of cardiac death, target-vessel MI, ischemia-driven TVR).	- Larger minimum stent area in OCT-guided PCI (5.72 ± 2.04 mm^2^) vs. angiography (5.36 ± 1.87 mm^2^), mean difference 0.36 mm^2^ (95% CI: 0.21–0.51, *p* < 0.001). - Lower stent thrombosis rate in OCT-guided PCI (0.5%) vs. angiography (1.4%), HR: 0.36 (95% CI: 0.14–0.91, *p* = 0.02). - Longer procedural time and higher contrast volume in OCT-guided PCI.
OCTOBER (2023)/1201 [52]	Multicenter, randomized, OCT- vs. angiography-guided PCI in complex bifurcation lesions.	MACE at 2 years (composite of cardiac death, target-lesion myocardial infarction (MI), and ischemia-driven TLR).	- Lower incidence of MACE in OCT-guided PCI (10.1%) vs. angiography-guided PCI (14.1%), HR: 0.70, 95% CI: 0.50–0.98, *p* = 0.035. - Lower stent thrombosis in OCT-guided PCI (0.5%) vs. angiography-guided PCI (1.4%), HR: 0.36, 95% CI: 0.14–0.91, *p* = 0.02.
OCTIVUS (2023)/2008 [55]	Prospective multicenter, randomized controlled trial, OCT-guided vs. IVUS—guided PCI in patients with significant coronary artery lesions.	TVF, a composite of Cardiac death, target vessel myocardial infarction, or ischemia-driven TVR.	- OCT was noninferior to IVUS (2.5% vs. 3.1%; absolute difference −0.6%, upper boundary 97.5% CI: 0.97%; *p* < 0.001 for non-inferiority). - Major procedural complications were lower in OCT vs. IVUS group (2.2% vs. 3.7%; *p* = 0.047).
ULTIMATE III (2024)/260 [48]	Randomized, IVUS-guided vs. angiography-guided DCB angioplasty in de novo coronary lesions.	In-segment LLL at 7 months.	- Lower LLL in IVUS-guided PCI (-0.10 ± 0.34 mm) vs. angiography-guided PCI (0.03 ± 0.52 mm), mean difference 0.14 mm, 95% CI: 0.02–0.26, *p* = 0.025. - Larger minimum lumen diameter at 7 months in IVUS-guided PCI (2.06 ± 0.62 mm) vs. angiography (1.75 ± 0.63 mm), *p* < 0.001. - Lower residual diameter stenosis in IVUS-guided PCI (28.15% ± 13.88%) vs. angiography (35.83% ± 17.69%), *p* = 0.001.
IVUS-ACS (2024)/3504 [50]	Multicenter, randomized, IVUS-guided vs. angiography-guided PCI in patients with acute coronary syndrome.	TVF at 1 year (composite of cardiac death, target vessel MI, or clinically driven TVR).	- Lower TVF in IVUS-guided PCI (4.0%) vs. angiography-guided PCI (7.3%), HR: 0.55, 95% CI: 0.41–0.74, *p* = 0.0001. - Reduction in target vessel MI: 2.5% (IVUS) vs. 3.8% (angiography), HR: 0.63, 95% CI: 0.43–0.92, *p* = 0.018. - Lower clinically driven TVR in IVUS-guided PCI (1.4%) vs. angiography (3.2%), HR: 0.44, 95% CI: 0.27–0.72, *p* = 0.0010. - Lower non-procedural MI in IVUS-guided PCI (0.6%) vs. angiography (1.5%), HR: 0.41, 95% CI: 0.20–0.84, *p* = 0.014.

## 7. Intravascular Imaging Versus Physiology

Although intravascular imaging and functional assessment are based in different physics and are two totally different modalities for CAD severity characterization, they share a mutual aim; to identify whether PCI would be beneficial or not for a specific lesion. Thus, several studies comparing intravascular imaging and functional assessment have been performed.

### 7.1. IVUS Versus FFR

Inherent limitations of IVUS pose problems in defining functional severity of lesions. According to a registry of 11 centers, 945 lesions were examined with both IVUS and FFR [74]. Combination of IVUS parameters did not improve prediction of functional significance. Minimal Lumen Area (MLA) and FFR demonstrated weak correlation (r = 0.289, *p* < 0.001). Independent prognostic factors of functional severity were male gender, left anterior descending (LAD) artery lesion, Left Ventricular Ejection Fraction (LVEF), lesion length, reference vessel diameter and MLA. Furthermore, several prognostic factors of anatomical and functional mismatch were identified: 17% of false positive cases were related to non-LAD lesions, while 24.4% of false negative cases with LAD lesions, Asian race and LVEF.

MLA may be used as an anatomic indicator of lesion severity. Best cut-off values appear to be 4 mm^2^ for non-LM and 6 mm^2^ for LM-lesions [75,76,77]. Takagi et al. first reported that IVUS-based MLA ≤ 3.0 mm^2^ (sensitivity, 83%, specificity, 92.3%) and area stenosis >0.60 (sensitivity,92.0%, specificity, 88.5%) predicted FFR ≤ 0.75 in 42 patients [78]. MLA showed a strong positive correlation with FFR values (r^2^ = 0.62, *p* < 0.0001), while area stenosis presented an inverse correlation with FFR values (r^2^ = 0.60, *p* < 0001). In the meta-analysis of Nascimento et al., MLA presented limited accuracy in predicting functionally significant lesions and led to misclassification in more than 1/5 of cases [79]. Moreover, investigators suggested lower cut-off values than the existing utilized in daily clinical practice for decision-making. It was the first attempt at data collection comparing IVUS with FFR. Similar results report Jang et al. in their meta-analysis with 4267 non-LM lesions [80].

Another important issue is that FFR may provide important information for lesion significance but not vulnerability. In a study of 13 lesions examined with FFR, OCT and hybrid Near-InfraRed Spectroscopy (NIRS)-IVUS, 11 lesions with FFR > 0.80 had characteristics of vulnerable plaque, i.e., thin-cap fibroatheroma, plaque burden > 70%, MLA < 4 mm^2^, or lipid core burden index > 100 [81,82].

Nam et al. conducted a comparative observational study which provided evidence that both FFR-guided and IVUS-guided PCI strategy for intermediate coronary artery disease were associated with favorable outcomes [83]. Interestingly, IVUS guidance led to a higher percentage of intervention than FFR guidance (91.5% in the IVUS- vs. 33.7% in the FFR-guided group, *p* < 0.001). The recent Fractional Flow Reserve and Intravascular Ultrasound—Guided Intervention Strategy for Clinical Outcomes in Patients with Intermediate Stenosis (FLAVOUR) study with 1682 patients illustrated the non-inferiority of FFR-guided with IVUS-guided PCI [84]. PCI was performed when FFR was ≤0.80 and in the IVUS-group when minimal lumen area ≤ 3mm^2^ or 3 to 4 mm^2^ combined with plaque burden more than 70%. In this study patients with intermediate stenosis in the coronary vessels were randomly allocated in two groups, one group undergoing IVUS-guided procedure and the second FFR-guided procedure. In the IVUS guided group a higher number of patients underwent revascularization (65.3%) in comparison with the FFR-guided group (44.4%). However, in both groups, the number of stents and their size used was similar. The incidence of MACE at 24 months, was 8.1% in the FFR group versus 8.5% in the IVUS group, with an absolute difference of 0.4% in favor of the FFR. (*p*-no-inferiority = 0.015).

Ahn et al. conducted an external validation of the prognostic value of post stenting FFR [85]. Taking into consideration from previous studies that post-stenting FFR may be a useful prognostic factor, with lower FFR values being correlated with suboptimal stent deployment and higher instance of adverse events, the investigators conducting a study where they utilized an IVUS strategy at the same time with post-stenting FFR. The intracoronary imaging-guided lesion preparation, stent sizing, and post dilation (iPSP) strategy included stent sizing and post dilation. Patients were divided in two groups, one receiving iPSP and the other not receiving (no-iPSP group). According to the results lower post-stenting FFR values were associated with higher target vessel failure in five years follow up in the overall population (per 0.01 increase in FFR, adjusted HR [aHR], 0.94; 95% CI: 0.90–0.98; *p* = 0.004) and in the no-iPSP group (per 0.01 increase in FFR, aHR: 0.94; 95% CI: 0.90–0.99; *p* = 0.009). On the contrary in the iPSP group no significant association was found between lower post-stenting FFR values in the prognosis of target vessel failure in the five years follow up (per 0.01 increase in FFR, aHR: 1.00, 95% CI: 0.96–1.05, *p* = 0.95). Additionally, in the patient population that underwent any form of imaging, the difference between pre-stenting FFR and post-stenting FFR (delta FFR) was significantly greater (0.18 ± 0.13 vs. 0.16 ± 0.11, *p* = 0.001), a finding that may suggest that improved physiological gain is supported by imaging modalities.

### 7.2. OCT Versus FFR

The correlation of OCT and FFR was first assessed in 2012 by Shiono et al. in 62 intermediate stenoses [86]. OCT parameters displayed good correlation with FFR. MLA < 1.91 mm^2^, Minimum Lumen Diameter (MLD) < 1.35 mm^2^ and percent lumen area stenosis > 70% were suggested as the best cut-off values for predicting FFR < 0.75. These values are smaller than values suggested in IVUS studies.

Pawlowski et al. suggest OCT as a useful method in adding information on lesion characteristics to FFR measurements [87]. In their registry of 71 lesions assessed simultaneously with OCT and FFR, the OCT-based MLA cut-off proposed to present good correlation with hemodynamically significant stenoses was 2.05 mm^2^ with accuracy 87%, sensitivity 75% and specificity 90% (*p* < 0.001). No significant relationship between FFR values and lesion length was documented. Similarly, another study revealed weak to moderate correlation of OCT variables (MLA, lesion length) and FFR value in intermediate stenoses [88]. Lesion morphological characteristics did not seem to play a major role in hemodynamic relevancy; still sample size was small and included only patients with stable coronary disease.

The first study comparing outcomes of OCT-guided versus FFR-guided PCI was conducted by d’ Ascenzo et al. [89]. They demonstrated that OCT-driven revascularization led to lower incidence of Target Lesion Revascularization (TLR) compared to FFR-driven revascularization (4.1% vs. 14.2% *p* < 0.01). No differences in all-cause death (3.6% vs. 1.1%, *p* = 0.34) and MACE (14.2% vs. 14.2%, *p* = 1) were identified between the groups. Still, this study was not randomized, and these results were based on propensity-matched analysis.

On the other hand, in the Fractional Flow Reserve vs. Optical Coherence Tomography to Guide Revascularization of Intermediate Coronary Stenosis (FORZA) trial, 350 patients with angiographically intermediate lesions were randomized in either OCT-guided or FFR-guided PCI [90]. PCI was performed in patients with FFR ≤ 0.80 and if area stenosis was ≥75% or 50% to 75% with minimal luminal area < 2.5 mm^2^ or plaque rupture. The occurrence of MACE in 13-month follow-up was 8.0% in the OCT group and 14.8% in the FFR group (*p* = 0.048). Interestingly, FFR led to more cases of medical management and lower total costs.

The recently published 5-year follow-up of the FORZA trial showed that the occurrence of MACE in the OCT group (16.7%) was still lower than in the FFR group (18.2%) but was statistically non-significant. (*p* = 0.704) [91]. These findings suggest that both technologies are effective in predicting future adverse events; however, imaging-based guidance might offer a relative advantage in more accurately forecasting events in the near term. A link between lesion complexity and guiding methods was showcased in subgroup analyses, with OCT being favored for complex lesions while FFR for non-complex lesions.

### 7.3. Intracoronary Imaging Versus iFR

There are few data in the literature considering the comparison of iFR with IVUS or OCT. Matsushita et al. were the first to evaluate the relationship between iFR and intracoronary imaging measurements [92]. Both FFR and iFR demonstrated satisfactory correlation with both FFR and OFDI. Interestingly, mismatch was noted in 20 lesions between FFR and iFR; FFR results were in favor of significant disease while iFR results were not, but not vice versa. This finding was probably associated with lesion location. Moreover, Yamazaki et al. reported mismatch between plaque vulnerability and functional severity using NIRS-IVUS [93]. No significant difference in vulnerability characteristics of lesions with FFR ≤ 0.80 and FFR > 0.80 was reported (26.2% vs. 29.4%). Since no randomized trials comparing IVUS or OCT with iFR exist, further studies are required.

### 7.4. QFR and Imaging

Zuo et al. tried to correlate QFR measurements and intravascular imaging modalities findings [94]. In this post hoc analysis, they aimed to associate QFR with IVUS-defined vulnerable plaque characteristics in patients with either Chronic Coronary Syndrome (CCS) or NSTEMI. The analysis obtained data from two prospective studies and included patients having undergone OCT or IVUS. According to QFR findings, the visualized lesions with OCT were classified in three groups: QFR-T1(QFR ≤ 0.85), QFR-T2 (0.85 < QFR ≤ 0.93) and QFR-T3(QFR > 0.93), while these with IVUS into two groups; low QFR (QFR ≤ 0.87) and high QFR (QFR > 0.87). In the QFR-T1 tertile, a higher number of OCT-defined Thin Cap Fibroatheroma (TCFA) was observed compared to the middle and the high tertile (*p* = 0.003 and *p* = 0.018, respectively). According to the multivariate analysis, QFR ≤ 0.80 was an independent determinant of OCT-TCFA and the Area Under the Curve (AUC) for the identification of OCT-defined TCFA lesions was 0.72 (*p* = 0.003). In the IVUS groups, the lower QFR group was associated with smaller minimal lumen area and greater plaque burden (*p* = 0.025 and *p* = 0.036, respectively). Nevertheless, no association between QFR and the IVUS virtual histology-defined TCFA or the composition of the plaques was found. (AUC = 0.54, *p* = 0.733). According to another retrospective analysis, composite IVUS parameters, including plaque burden and lesion length, showed a strong correlation with QFR ≤ 0.80. The combined IVUS model had an AUC 0.862, with a diagnostic accuracy of 84.8%, sensitivity 82.6% and specificity 72.5% [95].

FAVOR III Europe (Angiographic Quantitive flow ratio—guided coronary intervention) results showed that QFR did not demonstrate non-inferiority to FFR. The primary endpoint of MACE—death, MI, or unplanned revascularization at 12 months post randomization, occurred in more patients in the QFR group (6.7%) in comparison to the FFR group (4.2%). These results highlight the importance for the conduction of more randomized trials to examine the application of QFR in clinical settings [96].

FLAVOUR II a prospective, randomized, multicenter study, which included more than 1800 patients with hemodynamically significant stenoses and compare QFR or μQFR with IVUS guided decision for PCI [97]. In the FFR-group, 81% were guided by μQFR while in 19% of the patients QFR was used. At 12 months, the primary composite outcome of death, myocardial infarction, or revascularization occurred at similar rates in the FFR and IVUS groups (6.3% vs. 6.0%; HR 1.04, 95% CI 0.71–1.51; *p*-non-inferiority = 0.022). The FFR-guided group required fewer stents per patient (1.06 vs. 1.22, *p* < 0.0001) and achieved higher rates of optimal PCI, establishing non-inferiority of FFR-guided PCI compared to IVUS-guided PCI for clinical outcome and indicating a future shift in the treatment of cardiovascular disease.

## 8. Physiology and Imaging in Complex Settings

The implementation of both physiology evaluation and imaging seems to optimize plagues evaluation and PCI results. In the Synergy Between Percutaneous Coronary Intervention With TAXUS and Cardiac Surgery (SYNTAX II) study, a multicenter, prospective study with patients with multivessel disease, iFR/FFR- and IVUS-guided PCI showed beneficial results during one-year follow-up, emphasizing on the value of the performance of both methods [98]. The assessment of intermediate LM-stenoses using physiology modalities is not infrequent and is particularly challenging, due to the inability to use adenosine and the need to disengage the guiding catheter. In the largest trial so far only 23% of intermediated LM-lesions were classified as hemodynamically relevant and these patients proceeded to CABG [99,100]. Nonetheless, clinical outcomes at 5-year follow-up were similar in both surgical and non-surgical groups.

Moderate correlation between FFR and iFR was documented in other studies of LM. In the iLITRO-EPIC07 trial (Instantaneous Wave—Free Ratio for the Assessment of Intermediate Left Main Coronary Artery Stenosis: Correlations With Fractional Flow Reserve/Intravascular Ultrasound and Prognostic Implications) with 300 patients with intermediate LM disease, FFR and iFR demonstrated 80% agreement [101]. This trial was the first trial examining the discrepancy of the two functional methods in LM disease, to add further assessment with IVUS regarding whether to defer or proceed to PCI and to assess long-term outcomes in this specific population. In conclusion, the incidence of MACE at 20-month follow-up did not differ significantly between the group that undergone and deferred PCI (HR: 0.71, 95% CI: 0.30–1.72, *p* = 0.45), still IVUS could be a valuable tool in discordant cases. On the other hand, iFR has not been extensively validated in LM disease.

Considering clinical endpoints, Iannacone et al. performed the first meta-analysis comparing functional, imaging modalities with angiographic guidance in PCI [102]. Randomized Controlled Trials (RCTs) and propensity-score weight-matched studies were included. Clinical endpoints evaluated were MACE, target vessel revascularization (TVR), MI and stent thrombosis. IVUS was superior to angiography in terms of all clinical endpoints, while FFR in terms of MI and TVR (FFR OR: 0.74, CI: 0.57–0.99 for MI and OR 1.4, CI: 1.04–1.85 for TVR). OCT guidance reduced the incidence of TVR and stent thrombosis in comparison with angiography (OR 13.31; 1.62–67.2 for stent thrombosis and OR: 0.36 CI: 0.01–5.59 for TVR). Indirect comparison between functional and imaging modalities demonstrated comparable results, except of a trend towards a lower incidence of subsequent MI in favor of IVUS of OCT versus FFR in the context of ACS.

In patients with multivessel disease presented with ACS, functional imaging may play a significant role in optimizing management. The Complete Versus Lesion-Only Revascularization in Patients Undergoing Primary Percutaneous Coronary Intervention for STEMI and Multivessel Disease (CvLPRIT) trial evaluated complete revascularization during the index admission versus infarct-related–artery–only treatment [103]. Its findings indicated significantly reduced all-cause mortality and MACE with non–infarct-related artery treatment (*p* = 0.09 and *p* = 0.009, respectively), FFR guidance did not appear to provide additional benefit over angiography alone [104]. Furthermore, in a DANAMI 3-PRIMULTI sub-study (Primary PCI in Patients With ST-Elevation Myocardial Infarction and Multivessel Disease: Treatment of Culprit Lesion Only or Complete Revascularization), the use of FFR was associated with improved outcomes for the primary endpoint among patients with three-vessel disease (HR: 0.33, 95% CI: 0.17–0.64, *p* = 0.001), whereas no statistically significant difference was observed in those with two-vessel disease [105].

Despite strong recommendations for complete revascularization in this high-risk population, the optimal timing remains debated. In a meta-analysis by Panuccio et al., which pooled eight trials including 2256 hemodynamically stable STEMI patients with multivessel disease, immediate single-stage complete PCI during the index procedure was compared with deferred or staged complete revascularization [106]. No difference was observed in the primary composite of all-cause death, MI, and complete revascularization (RR: 0.95; 95% CI, 0.71–1.27), whereas cardiovascular death was higher with immediate complete revascularization (5.0% vs. 2.6%; RR: 0.39; 95% CI: 0.25–0.62, *p* < 0.01). Meta-regression indicated that drug-eluting stent use correlated with more favorable outcomes for the composite endpoint (*p* = 0.07). Accordingly, the timing of complete revascularization should be individualized based on clinical status, lesion complexity, and patient-specific characteristics.

In the FULL-REVASC trial (FFR-Guidance for Complete Nonculprit Revascularization), FFR-guided complete revascularization of nonculprit lesions during the index hospitalization was not superior to culprit-only PCI for the primary outcome at a median follow-up of 4.8 years (*p* = 0.53) [107]. By contrast, in the FIRE trial (Functional Assessment in Elderly MI Patients with Multivessel Disease), physiology-guided complete revascularization was associated with a lower incidence of the primary outcome in patients aged ≥75 years with MI and multivessel disease (15.7% vs. 21.0%; HR: 0.73, 95% CI: 0.57–0.93, *p* = 0.01) [108]. These contrasting results may be attributable to the longer follow-up in FULL-REVASC (4.8 years vs. 1 year) and differences in the enrolled populations. Further trials and analyses are warranted to clarify these observations. Regarding chronic total occlusions (CTO) imaging modalities including IVUS, OCT and CCTA, provide an efficient way to facilitate PCI in these complex procedures. IVUS has the ability to penetrate deeper, work without the use of contrast agents, and the real-time usage helps resolving the proximal cap ambiguity, sizing or optimizing stents and characterizing calcium nodules and vessels remodeling and finally guiding re-entry during retrograde or antegrade or dissection or reverse-CART strategies by confirming intraplaque versus subintimal wire positions [109,110,111]. OCT’s superior resolution makes it useful after stenting to detect edge dissections, malapposition, tissue protrusion, and to study healing, but its need for contrast and limited penetration restrict intra-procedural crossing guidance [67,70].

Coronary Computed Tomography Angiography (CCTA) can be useful in CTO procedure. According to a review conducted by Werner et al. computed tomography angiography (CTA) provides detailed preprocedural assessment of lesion characteristics including occlusion length, extend of calcification, presence of a calcified proximal cap, and evidence of negative remodeling [112,113]. Furthermore, the CT-RECTOR score (CT-Registry of Chronic Total Occlusion Revascularization), based on CTA and selected clinical variables, predicts 30 min antegrade wire crossing more accurately than the angiography-based J-CTO score. When the score exceeds 2, success rates fall from 90% to 66%, and drop further to 40% with higher values. Its key advantage over J-CTO is the ability to detect multiple lesions within the occlusion body, a feature often missed on angiography. The CTA-derived J-CTO score improves prediction of both wire-crossing time and overall procedural success, while the KCCT score (Korean Multicenter CTO CT Registry) forecasts procedural success and incorporates 2 additional parameters; a side branch at the occlusion entry and central calcification within the occlusion. Fusion of CTA with fluoroscopy allows real-time overlay of a CTA-derived vessel centerline, which aids identification of optimal viewing angles, guides antegrade dissection reentry maneuver, and supports guidewire advancement through long occlusions. CTA-guided PCI is most beneficial in long right coronary artery occlusions with little calcification and inconclusive location of the proximal cap or adjacent side branches, where it can help delineate vessel course and support guidewire navigation. Its main limitation is the spatial resolution of approximately 0.6 mm, which restricts reliable visualization of most collaterals, although large collaterals can sometimes be identified. Additional radiation exposure should be considered, but ongoing technical advances are improving image quality while reducing dose, and further refinements in fusion imaging may help define which lesion types benefit most from this approach.

To further evaluate the effectiveness of the scores, several studies were conducted. [114,115,116]. Panuccioc et al. conducted a study that included 140 patients with analyzable pre-procedural CCTA from a cohort of 217 elective CTO-PCI cases, of whom 28 (20%) had “full-moon” calcification (defined as a continuous 360° ring of calcium completely encircling the vessel lumen at the occlusion site on cross-sectional CCTA). In multivariable analysis adjusted for chronic kidney failure and prior coronary artery bypass graft surgery, this morphology was independently associated with higher odds of the primary endpoint, defined as failure of guidewire, microcatheter, or balloon crossing and/or the need for intravascular lithotripsy or rotational atherectomy (OR 6.5, 95% CI 2.1–20.5, *p* = 0.001), as well as lower procedural success (71.4% vs. 87.5%, *p* = 0.03), higher perforation rates (14.2% vs. 3.5%, *p* < 0.02), and longer procedural (172.5 vs. 144.0 min, *p* = 0.02) and fluoroscopy time (62.6 vs. 42.8 min, *p* = 0.03). MACE rates did not differ significantly between groups (3.5% vs. 0.8%, *p* = 0.29). Among different CTO complexity scores, the CCTA-based KCCT, which explicitly weights full-moon calcium, showed better discrimination for the primary endpoint (AUC 0.65, 95% CI 0.54–0.76, *p* = 0.01) than J-CTO (AUC 0.50, 95% CI 0.38–0.62, *p* = 0.96), Euro-CTO (AUC 0.47, 95% CI 0.36–0.58, *p* = 0.69), or CT-RECTOR (AUC 0.55, 95% CI 0.43–0.66, *p* = 0.39).

Exposure parameters in PCI is a major issue in clinical practice. A case–control study of 1047 patients showed that considering fluoroscopy time, FFR and IVUS displayed only a small effect and OCT no effect, and regarding contrast volume, FFR and OCT displayed only a small effect and IVUS no effect [117]. Radiation did not differ among these modalities. The number of stents implanted was the only parameter that significantly influenced exposure parameters.

## 9. Imaging-Derived Physiology Indices

In the past few years novel techniques have been proposed and tested based on the calculation of physiology properties derived from non-invasive (CT) and invasive (IVUS, OCT) methods.

### 9.1. IVUS-Based FFR

Bezerra et al. tried to implement FFR properties in an IVUS catheter, showing promising results [118]. IVUS-based FFR demonstrated overall accuracy, sensitivity, and specificity of 91%, 89% and 92%, respectively. This hybrid method utilizes a single catheter so as to acquire the IVUS images and with the assistance of deep learning methods the lumen boundaries are defined. The catheter route is predefined by coronary angiography images based on a graph theory algorithm and combined with the images it constructed a 3D mode of the vessel. To estimate blood flow rate, TIMI frame count and a fluid dynamics equation are used. This method performed better than IVUS-based MLA or angiography in detecting significant lesions. Similar findings are reported by Seike and his colleagues; IVUS-derived FFR correlated better with FFR (R = 0.78, *p* < 0.001) than with IVUS-derived MLA (R = 0.43, *p* = 0.002) [119]. Higher values were reported by Yu et al. in 167 paired comparisons: the diagnostic accuracy, sensitivity and specificity, for IVUS-derived FFR to detect hemodynamically relevant lesions was 92%, 91% and 96%, respectively, with low inter- and intra- observer variability and regardless of lesion location, prior MI or type of catheter [120].

Additionally, apart from the calculation of FFR based on geometric characteristics of the vessel, another method was based on basic fluid dynamics. In the PROSPECT trial (Impact of Intravascular Ultrasound—Derived Lesion- Specific Virtual Fractional Flow Reserve Predicts 3- year Outcomes of Untreated Nonculprit Lesions), Seike et al. analyzed 3227 non-culprit, untreated lesions in patients with ACS with gray-scale and virtual histology IVUS and calculated an IVUS-derived FFR based on fluid dynamics [121]. Moreover, slight reduction calculated with this method in the long-term follow-up was introduced as an independent prognostic factor of future non-culprit MACE.

### 9.2. OCT-Based FFR

Attempts to use anatomical information from OCT, to identify the hemodynamic significance of a lesion have been made. This strategy allows imaging properties of plaque can be assessed as well. A study of 26 lesions has confirmed a moderate correlation between pressure-derived FFR and OCT-derived FFR (r = 0.69, *p* < 0.001) [122]. There are reports about OCT-based virtual FFR, using vessel reconstruction, demonstrating very high correlation with pressure-derived FFR [123].

Huang et al. investigated optical flow ratio, which was OCT-based, and quantitative flow ratio, which was angiographically based in 212 vessels [124]. OFR can be calculated fast, without need for induced hyperemia. Optical flow ratio performed better than quantitative flow ratio and had an overall accuracy, sensitivity and specificity in detecting lesions with FFR < 0.80 92%, 86% and 95%, respectively. Interestingly, prior MI or stenting did not affect the performance of this method. This novel method displayed good accuracy and low interobserver variability [125].

Worth mentioning is that OCT seems to play a major role not only in periprocedural decision-making but, also, in improved PCI outcomes The DOCTORS (Does Optical Coherence Tomography Optimize Results of Stenting) trial showed that, compared with angiography-guided PCI, OCT-guided PCI resulted in improved post-PCI FFR (0.94 vs. 0.92, *p* = 0.005) [64].

### 9.3. OCT-μFR

OCT-μFR is a novel computational technique combining both physiological assessment and high-resolution morphological assessment. This computational technique is based on angiographic and OCT images. μQFR (Murray low based QFR) is calculated from a single angiographic projection with several steps including delineating the lumen contours of the interrogated vessel and its side branches, reconstruction of the reference vessel’s diameter based on Murray’s law, TIMI frame count estimation of contrast flow velocity and calculation of pressure drop with fluid dynamics. Then in combination with OCT-acquired pullback images, it precisely delineates lumen contours and side branches. A new geometric model of the vessel is constructed and combined with OCT’s precise lumen measurements and accounting for the media size and step-down phenomenon on bifurcations, a new reference vessel diameter is quantified. Pressure drop is then re-calculated based on fluid dynamics [126].

According to a retrospective single-center observational study conducted by Xu et al. [126]. OCT-μFR was compared with μQFR in 269 vessels from 218 patients. The middle value of FFR was 0.81 ± 0.11 and FFR was ≤0.80 in 45% of the vessels. OCT-μFR was found to be more compatible with FFR (r = 0.830) in comparison to μQFR (r = 0.76) with a statistically significant difference (*p* = 0.018). The diagnostic performance of OCT-μFR was higher ([AUC] = 0.95) than that of μQFR ([AUC] = 0.92) but it was not statistically significant (*p* = 0.057). For the identification of stenosis that cause ischemia there was 89.3% sensitivity, 93.2% specificity, 91.5% positive predictive value, 91.4% negative predictive value, 13.2 positive likelihood ratio and 0.1 negative likelihood ratio. The presence of deficient angiographic images did not affect the ability of OCT-μFR in predicting FFR ≤ 0.80 ([AUC] = 0.94 vs. 0.93, *p* = 0.789) but it did affect the ability of μQFR ([AUC] = 0.94 vs. 0.87, *p* = 0.090). As for ideal angiographic images quality the diagnostic performance was comparable in both methods ([AUC] = 0.94 for μQFR vs. 0.94 for OCT-μFR, *p* = 0.879). For the estimation of tandem lesions OCT-μFR diagnostic performance was superior to μQFR([AUC] = 0.94 vs. 0.87 *p* = 0.017) but comparable in vessels without tandem lesions ([AUC] = 0.94 vs. 0.94, *p* = 0.977). In 54 interrogated vessels, OCT image pullbacks were unsuccessful to cover all lesions, with an FFR of 0.74 ± 0.14. As a result, OCT-μFR had lower AUC and diagnostic accuracy in populations with complete vs. incomplete lesion coverage (0.95 vs. 0.91, *p* = 0.386 and 93% vs. 87%, *p* = 0.196, respectively) but it was not statistically significant. Concerning bifurcation or no bifurcation lesions, OCT-μFR’s diagnostic performance was not significantly different([AUC] = 0.93 vs. 0.95, *p* = 0.479) and for patients with or without prior MI ([AUC] = 0.94 vs. 0.95, *p* = 0.944). OCT-μFR and μQFR had comparable diagnostic performance with bifurcation lesions ([AUC] = 0.93 vs. 0.91, *p* = 0.441) and in patients with prior MI ([AUC] = 0.94 vs. 0.93, *p* = 0.313).

### 9.4. OCT Derived Virtual Flow Reserve (VFR)

FUSION (Validation of OCT- Based Functional Diagnosis of Coronary Stenosis), a single-arm, prospective, multicenter, observational study compared Virtual Flow Reserve (VFR), an OCT derived method to invasive FFR [127]. Specifically, 312 patients with intermediate angiographic stenosis (40–60%) and presented with stable ore unstable coronary syndrome were enrolled and OCT-derived VFR was compared to invasive FFR. The average diameter stenosis as acquired from angiography was 65.5% ± 14.9%, the mean lesion length was 19.4 ± 9.3 mm and the diameter of the reference vessel was 3.2 ± 0.5 mm. The median FFR values were 0.83 ± 0.11 indicating that 38.4% of the patients exhibited FFR values < 0.80. The mean VFR was 0.81 ± 0.12 with 41.4% of the patients exhibiting VFR value 0.80 or lower. Using a binary cutoff of 0.80, the accuracy of the method was 82%, the sensitivity 80.4%, the specificity was 82.9%, the positive predictive value was 74.5% and the negative predictive value of VFR was 87.2%.

## 10. Discussion

The assessment of CAD has evolved significantly with the development of physiological and anatomical modalities. Angiography remains the cornerstone of imaging, though the modality itself is unable to assess the functional significance, demanding more advanced technologies. Each modality holds its benefits and limitations, influencing clinical decision-making and the outcomes related to the patient and the choice between them revolves around their application and usefulness in clinical applications.

FFR and NHPIs provide real-time hemodynamic information. The ability of a lesion to cause ischemia can be determined at the time of imaging and thus it is ensured that interventions target significant blockages. Studies, such as the FAME trials, have demonstrated superior outcomes in FFR-guided PCI compared to angiography alone leading to a reduction in unnecessary stenting and the improvement of patient prognosis [25,26,27,28]. However, studies like FUTURE and RIPCORD-2 showed that FFR was unable to provide a significant benefit in the quality of life of and cardiovascular outcomes of the patients in comparison to angiography alone outcomes that need to be further evaluated in future clinical studies [30]. A recent meta-analysis by Sánchez et al. showed that FFR-guided PCI in comparison with angiography-guided PCI led to reduction in all-cause mortality and MI (OR: 0.79, 95% CI: 0.64–0.99, I^2^ = 53% and OR: 0.74, 95% CI: 0.59–0.93, I^2^ = 44.7%, respectively) but there was no significant difference between the two groups regarding the major cardiac adverse events (OR: 0.86, 95% CI 0.72–1.03, I^2^ = 72.3%) [128]. Large scale trials investigating these results are needed to further investigate these results and clarify the applicability of FFR in clinical practice. Contemporary studies support complete revascularization in ACS with multivessel disease, but the value of physiology and optimal timing remain uncertain. CvLPRIT and FULL-REVASC were neutral for FFR guidance, while FIRE and DANAMI-3-PRATI suggest that there is a benefit in FFR utilization in specific patient populations, specifically in older patient populations and in three vessel disease. It is of outmost importance that decisions for staged or single stage strategies should be individualized to clinical status, lesion complexity, and patient profile rather than applied uniformly.

IVUS and OCT provide high resolution visualization of plaque burden, vessel size, and luminal changes, improving PCI outcomes. A recent meta-analysis showed that the utilization of IVUS in clinical practice leads to the reduction in major cardiac adverse events (*p* < 0.001), stent thrombosis (*p* = 0.046), TLR (*p* = 0.01), TVR (*p* < 0.001), results that support the use of IVUS in clinical practice [129]. Unlike physiology modalities that assess ischemic burden, imaging modalities estimate the characteristics of a lesion, providing insights into future events and guiding stent optimization, leading to better procedure outcomes. RENOVATE-COMPLEX and ILUMIEN-IV trials have established the benefits of imaging-guided revascularization, reducing the adverse events especially in patients with complex lesions [47,53,130]. Those come in accordance with the OCTOBER outcomes, where OCT guidance was found superior for the detection of complex bifurcation lesions in comparison with conventional angiography. When combined with optimal dual antiplatelet therapy duration, these strategies may contribute to favorable outcomes in this high-risk population [131,132,133,134]. FFR and NHPIs are superior in defining the functional significance of a lesion, at the time of the procedure while IVUS and OCT reduce possible complications such as stent thrombosis and restenosis.

Sex-related disparities in the use of functional and intracoronary imaging modalities may exist in contemporary clinical practice, potentially contributing to suboptimal care in female patients. A recent retrospective analysis including 1,454,121 patients demonstrated that female sex was an independent negative predictor for the use of Fractional Flow Reserve (FFR) [135] Specifically, FFR was performed significantly less often in women compared with men (OR: 1.2152; 95% CI: 1.1945–1.2361; *p* < 0.005). Female patients were also significantly older (*p* < 0.005), and among those presenting with acute coronary syndrome (ACS), FFR was utilized less frequently in women (OR: 0.827; 95% CI: 0.811–0.842; *p* < 0.001). A discussion exists regarding potential sex-based differences in FFR values. A sub-analysis of the FAME trial investigating sex differences in FFR revealed that women tend to have significantly higher FFR values than men (0.75 ± 0.18 vs. 0.71 ± 0.17; *p* < 0.01), and the proportion of functionally significant stenoses was lower in women for both intermediate (50–70%) and severe (70–90%) angiographic stenosis categories (*p* < 0.001 and *p* = 0.019, respectively) [136]. These higher FFR values in women have been attributed, in part, to microvascular dysfunction, as previously shown in the Women’s Ischemia Syndrome Evaluation (WISE) study [137]. Nevertheless, further sex-specific research is warranted to better understand these physiological differences and to evaluate whether sex-adjusted thresholds for FFR interpretation may be justified.

Moreover, an evaluation of sex related differences in the utilization of intracoronary imaging was conducted by Rashid et al. [138]. Among 994,478 patients, only 20,183 of 255,862 women (7.9%) underwent intracoronary imaging, compared to 8.4% of men, a difference that was statistically significant (*p* < 0.001). Female sex was once again identified as an independent negative predictor of intracoronary imaging use (OR: 0.93; 95% CI: 0.91–0.96). This underutilization persisted even in clinical scenarios where imaging is strongly recommended, such as acute coronary syndromes, long lesions, multivessel disease, chronic total occlusion, and left main coronary artery disease, suggesting a persistent sex-based disparity in procedural decision-making. These findings enhance the need for critical evaluation of current practices and advocate for the development of more sex-specific strategies in cardiovascular care. Addressing these disparities may help mitigate avoidable adverse outcomes and contribute to more equitable and effective management of female patients [139,140]. Despite their different aspects and applications, these modalities may be complementary rather than mutually exclusive. FLAVOUR trials have made a comparison of IVUS-guided versus FFR-guided PCI and found non-inferior outcomes, meaning that both modalities can be used to achieve the best outcome [141]. In real-world practice, a hybrid approach can be advantageous and provide the best outcomes for the patient.

The integration of both physiology and imaging modalities represents the next frontier in the optimization of PCI. Hybrid technologies such as IVUS-derived FFR or OCT-derived VFR utilize computational outcomes and fluid dynamics to estimate physiological significance using imaging modalities. Higher accuracy without the need for hyperemic agents and pressure wires has been presented in defining plaque severity. More research needs to be performed to further evaluate their efficacy and application in clinical settings, but their implementation in clinical practice may revolutionize and advance treatment.

## 11. Conclusions

Coronary angiography remains the standard for managing established coronary disease, while intravascular imaging and functional modalities enhance lesion assessment and guide decision-making. IVUS and OCT offer complementary diagnostic insights, while FFR and NHPIs assess hemodynamic significance. Current evidence supports the use of both approaches, with no clear superiority. Further research is needed to define their roles in ACS, left main disease, multivessel disease, and CABG planning. Emerging non-invasive techniques and combined imaging–physiology protocols may optimize care and improve outcomes.
